# Whole lifecycle observation of single‐spore germinated *Streptomyces* using a nanogap‐stabilized microfluidic chip

**DOI:** 10.1002/mlf2.12039

**Published:** 2022-09-24

**Authors:** Dongwei Chen, Mengyue Nie, Wei Tang, Yuwei Zhang, Jian Wang, Ying Lan, Yihua Chen, Wenbin Du

**Affiliations:** ^1^ State Key Laboratory of Microbial Resources, Institute of Microbiology Chinese Academy of Sciences Beijing China; ^2^ College of Life Sciences University of the Chinese Academy of Sciences Beijing China; ^3^ Savaid Medical School University of the Chinese Academy of Sciences Beijing China; ^4^ Qingdao Institute of BioEnergy and Bioprocess Technology Chinese Academy of Sciences China

**Keywords:** microfluidics, multicellular differentiation, single cell analysis, single‐spore germination, *Streptomyces*

## Abstract

*Streptomyces* is a model bacterium to study multicellular differentiation and the major reservoir for antibiotics discovery. However, the cellular‐level lifecycle of *Streptomyces* has not been well studied due to its complexity and lack of research tools that can mimic their natural conditions. In this study, we developed a simple microfluidic chip for the cultivation and observation of the entire lifecycle of *Streptomyces* development from the single‐cell perspective. The chip consists of channels for loading samples and supplying nutrients, microwell arrays for the seeding and growth of single spores, and air chambers beside the microwells that facilitate the development of aerial hyphae and spores. A unique feature of this chip is that each microwell is surrounded by a 1.5 µm nanogap connected to an air chamber, which provides a stabilized water–air interface. We used this chip to observe the lifecycle development of *Streptomyces coelicolor* and *Streptomyces griseus* germinated from single spores, which revealed differentiation of aerial hyphae with progeny spores at micron‐scale water–air interfaces and air chambers. Finally, we demonstrated the applicability of this chip in phenotypic assays by showing that the microbial hormone A‐Factor is involved in the regulatory pathways of aerial hyphae and spore formation. The microfluidic chip could become a robust tool for studying multicellular differentiation, single‐spore heterogeneity, and secondary metabolism of single‐spore germinated *Streptomyces*.

## Impact statement

We describe a microfluidic chip that mimics the natural porous environment for the growth and development of *Streptomyces*, a central paradigm for the study of bacterial multicellularity and natural product discovery. We uesd a microfluidic chip for single‐celled spore germination, cultivation, and observation of the whole lifecycle of *Streptomyces*, including the development of aerial hyphae and chains of spores. In addition, we also observed the early growth, opportunistic transformation, and anastomosis of aerial hyphae of *Streptomyces* with precise time and stimulation control. We believe this microfluidic‐based approach will play an essential role in understanding the complex multicellular differentiation and the regulation of secondary metabolites of *Streptomyces*.

## 1 INTRODUCTION

*Streptomyces* is a genus of filamentous bacteria that play crucial roles in various habitats with their broad range of metabolic and biochemical processes, including degradation of chitin and cellulose^1^, ^2^, ^3^. They are the most important natural source of bioactive compounds, such as antibiotics and antitumor agents, producing two‐thirds of the antibiotics of medical and agricultural interests^4^, ^5^, ^6^. In their natural conditions, *Streptomyces* grows at air–liquid–solid interfaces in soil within porous structures that retain water in micron‐sized cavities and channels. Nutrients, oxygen, and water transport, and other environmental factors profoundly impact their physiology, morphological development, and secondary metabolism^7^. Recent research with advanced genetic tools has made significant progress in uncovering the physiological and metabolic potential of *Streptomyces* for natural products. However, many cryptic secondary metabolite pathways of *Streptomyces* remain either silent or poorly expressed for cells grown on agar plates or in liquid media in standard laboratory conditions, presumably due to the inability to recreate nutritional and environmental conditions in their natural soil habitat.

Microfluidics has emerged as a new tool to study microbes, offering many advantages, such as micrometer‐scale spatial resolution and flexible temporal control of nutrient exchange and chemical gradients^8^. Microfluidic techniques have been used to study microbiology in many ways^9^, ^10^, such as single‐cell isolation and cultivation^11^, bacterial chemotaxis^12^, quorum sensing^13^, and population dynamics^14^. Although high‐throughput enrichment and sorting of soil‐derived *Actinobacteria* in microfluidic droplets have been described^15^, microfluidic devices that allow the development and differentiation of *Streptomyces* are rarely reported. The challenge in *Streptomyces* cultivation is that their growth and differentiation rely on a stabilized water–air interface. When cultivated on solid agar, *Streptomyces* has a differentiated lifecycle with precisely controlled stages, including germination of vegetative hyphae in the substrate, formation of hydrophobic aerial hyphae, and development of airborne spores that allow dispersion^16^. However, in a standard liquid medium, *Streptomyces* mainly exists as vegetative hyphae that tangle together to form many small pellets and clumps with very few aerial hyphae^17^. Therefore, direct miniaturization of standard liquid culture is not an ideal approach for studying the development of *Streptomyces*.

To overcome these challenges, we describe a microfluidic chip integrating liquid containing microwells and air chambers to establish a stabilized water–air interface for cultivation, the whole lifecycle observation of *Streptomyces* differentiation, and phenotypic assay. The chip can achieve micron‐scale spatial resolution, maintain long‐term culture conditions, initialize time‐dependent chemical exchange, and enable single‐cell cultivation and observation. Thus, the chip is a versatile tool for exploring the development and behavior of *Streptomyces* under well‐controlled circumstances. We evaluated the chip's performance by single‐cell cultivation of two model representative *Streptomyces* strains. Moreover, we performed a precisely controlled phenotypic assay with A‐Factor analog *β*‐keto SCB2, which is involved in autoregulation of secondary metabolism and morphological differentiation in Actinomycetes^18^.

## 2 RESULTS

### 2.1 Design of the microfluidic chip

**Figure 1:**
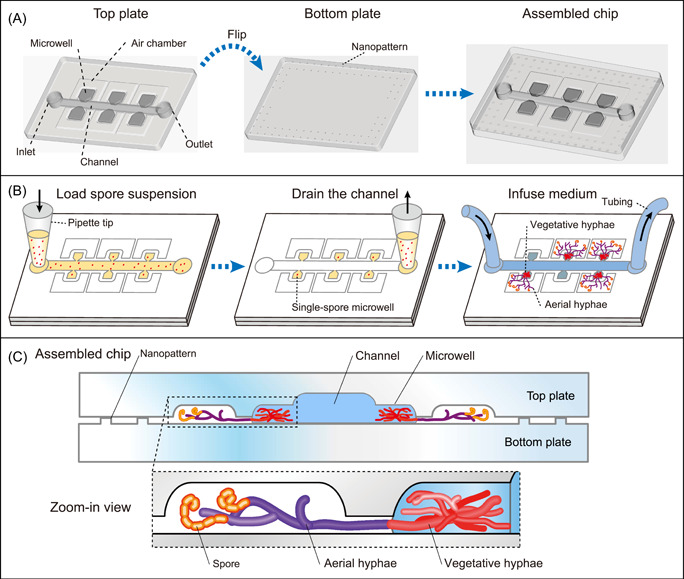
Illustration of the microfluidic chip for lifecycle observation of *Streptomyces*. (A) Schematic diagram of assembly and setup of the microfluidic chip. The dimensions of the microfluidic chip are shown in Supporting Information: Materials. (B) Spore suspension is loaded into the microwells by a pipette. The concentration of spores is controlled to allow single spore trapping in the microwells based on a Poisson distribution. The channel was drained by a pipette to remove extra spores. The culture medium was continuously infused into the chip to allow the whole lifecycle development process. (C) Schematic of the sectional view of the chip with *Streptomyces* lifecycle development shown in a zoom‐in view.

**Figure 2:**
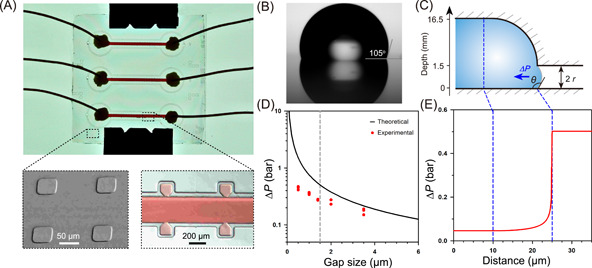
Characterization of the microfluidic chip with nanogap‐stabilized liquid–air interfaces. (A) A picture of the assembled chip. There are 40 microwells symmetrically distributed along the channel with three parallel replicates. The 1.5‐µm height nanopatterns on the bottom plate are observed via scanning electron microscopy. The chip was assembled and filled with red dye, as shown in the zoom‐in view. (B) The silanized glass plates of the chip have a contact angle of 105° with deionized water. (C) A side view of the water–air interface between the microwell and the gas chamber shows the direction of liquid surface tension at the microwell edge. (D) Relationship between surface tension and gap size at the water–air interface. (E) The surface tension distribution along the microwell.

We designed a microfluidic chip with an array of microwells for the entire lifecycle observation of *Streptomyces* (Figures 1 and 2A). The chip incorporates two essential design features: (i) a stable water–air interface enabled by nanogaps between the microwells and air chambers; (ii) well‐controlled nutrient and chemical exchange through the main channel. We mimicked the natural habitat of *Streptomyces* by creating liquid‐containing microwells bridged with air chambers via nanogaps for vegetative and aerial growth, respectively (Figure 1A). The glass plates exhibited a hydrophobic surface after fluorinated silanization (Figure 2B–E). The nanogap between two assembled glass plates is 1.5 μm, generated by etched nanopatterns of the bottom plate. Stable water–air interfaces can form at the nanogap edge upon pipette‐filling of the microwells. As a result, the high surface tension of the liquid–air interface at the nanogap ensures long‐term observation without deleterious drift or shift, and the aerial hyphae (1 μm) can readily pass through the gap (Figure 1C). The chip contains 120 microwells symmetrically distributed along three parallel channels for observation of multiple single‐spore events, which facilitates the study of cellular heterogeneity (Supporting Information: AutoCAD design).

We regard the liquid surface as a spherical surface so that the capillary pressure Δ*P* can be derived from the following equation:

(1) $$\Delta P=\frac{2\sigma \cos\theta }{r}$$

Where *σ* is the liquid surface tension (7.28 × 10^−2^ N/m); *θ* is the contact angle between the liquid surface and the solid plate (the maximum value is 105°) (Figure 2B); and the radius (*r*) equals one‐half of the gap height (0.75 µm) (Figure 2C). Thus, the capillary pressure Δ*P* is calculated to be 5.03 × 10^4^ Pa, which is large enough to form a stable gas–liquid interface (Figure 2D,E). *Streptomyces* spores were appropriately diluted and loaded into microwells to achieve single spore isolation in microwells following a Poisson distribution. Spores can germinate, form vegetative hyphae in microwells, pass through the nanogap, differentiate into aerial hyphae in air chambers, and eventually develop into mature spores. The lifecycle of *Streptomyces* can last for several days, and thus we infused culture media continuously from the channel to guarantee adequate nutrient supply. The mycelia would not be disturbed because of the narrow joint between the channel and the microwells. The entire developmental process could be monitored using an inverted microscope.

### 2.2 On‐chip lifecycle observation of *Streptomyces coelicolor*

*S. coelicolor* is a model organism of *Streptomyces*, and the complete genome of the type strain *S. coelicolor* M145 has been sequenced; it is used in many studies of *Streptomyces* growth and development^2^. We cultivated *S. coelicolor* in the minimal medium on the chip and observed its entire lifecycle (Figure S1 and Movie S1). After 9 h of dormancy, the spore emerged from one germ tube, which prolonged and formed branches. Each branch showed apical growth, indicating that the group of cells grew at an exponential phase in the microwell. The hyphae could spread randomly in the liquid medium because there was no solid substrate confinement. The hyphae gradually approached the water–air interface, broke the surface tension, and grew into the air chamber at 28 h (growth almost perpendicular to the edge of the microwell). The aerial hyphae progressively elongated and formed branches in all directions. There were curls and spirals at the end of the hyphae. Meanwhile, the vegetative hyphae developed many layers and eventually almost filled the entire microwell. The vegetative and aerial hyphae stopped growing after 60 h (Movie S1).

When cultivated in a flask‐scale liquid medium, aerial hyphae formation and sporulation are blocked in most *Streptomyces* strains^19^, but when cultured in bioreactors, some strains may be able to sporulate due to stress conditions such as strong agitation^20^. It has been suggested that nutrient depletion and the reuse of materials led to the hyphae differentiation in liquid medium^21^, and that programmed cell death also triggered the differentiation process in liquid and solid media^17^. Although the specific signals are unclear, *N*‐acetylglucosamine produced by the decomposition of peptidoglycan may be one of the signals^22^. However, single‐cell whole‐lifecycle development has not been observed before. In this study, we cultivated *S. coelicolor* in the chip and found that vegetative hyphae did not lyse; instead, they continually grew even after the emergence of aerial hyphae. Furthermore, the culture medium was supplied continuously into the chip such that the nutrient depletion did not occur, indicating that the differentiation phenomenon may not necessarily be correlated with nutrient depletion.

### 2.3 On‐chip differentiation of *S. coelicolor* in yeast extract‐malt extract (YEME) medium

**Figure 3:**
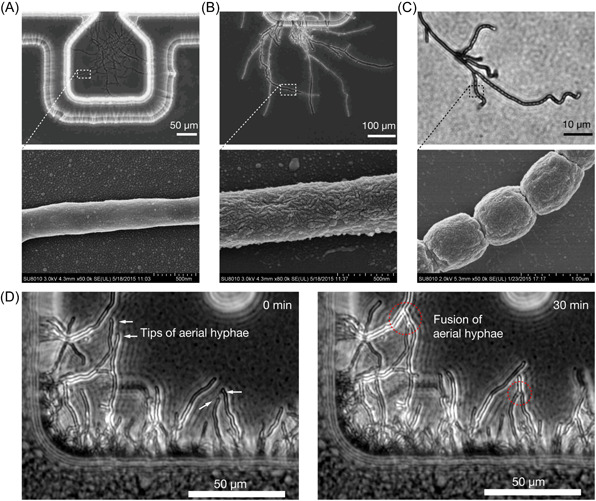
Development of *Streptomyces coelicolor* cultivated in a microfluidic chip. (A–C) The vegetative hyphae (A), aerial hyphae (B), and spores (C) of *S. coelicolor* were observed by optical microscopy (top panel) and electron scanning microscopy (bottom panel), respectively. (D) Time series imaging of hyphal anastomosis (fusion) in *S. coelicolor*. Some hyphal tips (arrows) were growing toward a hyphal peg for subsequent fusion.

*S. coelicolor* can form aerial hyphae and spores in standing liquid cultures with minimal media but not rich media^23^. Here, we inoculated single spores in microwells with a nutrient‐rich YEME medium and cultivated the samples for several days to test whether they could differentiate (Figure 3). The results showed that *S. coelicolor* still had a complete lifecycle in liquid YEME medium, including vegetative hyphae in microwells (Figure 3A) and aerial hyphae in the air chambers (Figure 3B) with spiral spore chains on the aerial hyphae (Figure 3C). Scanning electron microscopy (SEM) revealed that the hyphae in the microwells had a relatively smooth surface (Figure 3A). Aerial hyphae in the air chamber had a layer of well‐organized hydrophobic proteins^24^ (Figure 3B). The mature spores formed spiral chains with compartments between each spore (Figure 3C). These results are consistent with the development of *S. coelicolor* grown on solid plates and previous reports on the microscopic features of hydrophobic proteins^25^.

Accordingly, *S. coelicolor* had entire lifecycles in the liquid environment regardless of the nutrient status. An earlier study showed that the expression of most genes is comparable between liquid and solid cultures, including genes involved in the hydrophobic cover formation and even a few genes regulating the early stages of sporulation^26^. Genes involved in the final stages of hydrophobic cover/spore maturation are upregulated in solid cultures compared with liquid cultures. These findings suggest that *S*. *coelicolor* can differentiate in both solid and liquid cultures. Transcripts and proteins are ready before aerial hyphae formation. Once *S. coelicolor* senses the existence of air, they grow aerial hyphae and develop into mature spores. In standing liquid cultures, a physical constraint may hinder aerial hyphae formation. A nutrient‐rich medium contains more complex ingredients, which are likely to attach to the hyphae surface and reduce the hydrophobicity of the hyphae, making it difficult for the aerial hyphae to erect through the liquid–air interface.

Interestingly, we observed the merging of aerial hyphae when we cultivated *S. coelicolor* in YEME (Figure 3D and Movie S2). Two hyphal tips grew toward each other until contacted and fused. We also recorded hypha‐to‐peg or hypha‐to‐side fusion, as a hyphal tip approached the side of another existing hypha and merged with it. This universal phenomenon in Streptomycetes is called hyphal anastomosis or hyphal fusion, which was first confirmed in *S. scabies* ^27^, and is considered to be very important for intrahyphal communication, nutrient/water translocation, and general homeostasis within a colony^28^.

### 2.4 On‐chip observation of wild‐type *Streptomyces griseus*

Next, we applied the chip to cultivating another model organism *S. griseus*, to investigate its differentiation in liquid cultures. We cultured *S. griseus* in liquid minimal medium (MM) and YEME medium, respectively, and observed its three lifecycle stages through optical microscopy and electron microscopy (Figure S2). The results confirmed that *S. griseus* could accomplish its whole lifecycle in both liquid cultures, with the exact differentiation mechanism as that on solid plates. Furthermore, we found that the growth of aerial hyphae and sporulation did not rely on the lysis of vegetative hyphae, suggesting that genes encoding extracellular proteases and protease inhibitors may not be necessary for the morphological differentiation of *S. griseus*.

### 2.5 Phenotypic recovery of *S. griseus* Δ*afsA* mutant with A‐Factor analog

A‐Factor is the master switch for morphological differentiation and secondary metabolism in *Streptomyces* ^29^. For *S. griseus* growing on the solid plate, the differentiation process begins with the expression of *afsA* that controls the synthesis of A‐Factor^29^. The binding of the A‐Factor with its receptor protein, ArpA, relieves the suppression of *adpA* by ArpA. Afterward, AdpA stimulates aerial hyphae growth, spore development, and secondary metabolism by regulating various genes, including *ssgA* ^30^, *adsA* ^29^, *amfR* ^31^, extracellular proteases^32^, ^33^, and protease inhibitor encoding genes^34^. Hitherto, the effect of A‐Factor on the differentiation of *S. griseus* on solid agar and liquid medium has not been studied due to the inability to maintain a stabilized liquid–air interface to support aerial hyphae development.

**Figure 4:**
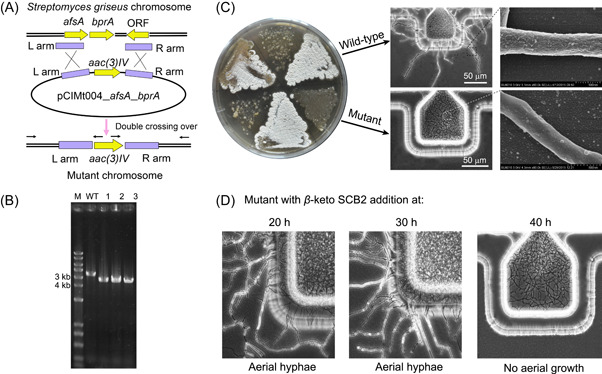
The role of the A‐Factor in the development of *Streptomyces griseus*. (A) Illustration of *S*. *griseus* Δ*afsA* mutant construction. (B) Electrophoresis of PCR products of wild‐type (WT) *S. griseus* and its Δ*afsA* mutants. (C) Phenotypes of *S. griseus* wild‐type and Δ*afsA* mutant on a solid plate. After cultivation on the chip, we observed aerial hyphae of *S. griseus* wide‐type and Δ*afsA* mutant via optical microscopy and SEM. (D) The feeding of the A‐Factor analog recovered aerial growth of Δ*afsA* mutant at 20 and 30 h after cultivation, but no effect was observed at 40 h.

We constructed an *S. griseus* Δ*afsA* mutant via genetic engineering that could not form aerial hyphae on YEME agar (Figure 4A, B). We inoculated the mutant onto YEME agar and cultivated it for several days. Compared with the wild type, the mutant could not develop either aerial hyphae or pigmented spores on the agar (Figure 4C). The parallel on‐chip cultivation revealed that the vegetative hyphae formed mainly in medium‐filled microwells with very few hyphae outside microwells, which were very short even after being cultivated for several days and could not form spores. SEM images showed that the hyphae surface of *S. griseus* Δ*afsA* mutant was relatively smooth, indicating that these short hyphae were still vegetative hyphae. Therefore, the phenotype of *S. griseus* Δ*afsA* mutant grown in chip‐based culture was consistent with that grown on the solid plate.

Next, we synthesized an A‐Factor analog *β*‐keto SCB2 and fed it at different time points to the Δ*afsA* mutant to examine whether it could recover its differentiation phenotype. Previous studies showed that the production of the A‐Factor is growth‐dependent^35^. A‐Factor accumulates during vegetative growth, reaches a peak concentration of 25–30 ng/ml, and rapidly decreases thereafter^35^. As shown in Figure 4D, the mutant formed aerial hyphae and spores when we added *β*‐keto SCB2 at 20 and 30 h after inoculation. The SEM imaging confirmed the existence of hydrophobic proteins on the surfaces of aerial hyphae and spores of Δ*afsA* mutant when we fed *β*‐keto SCB2 at 30 h (Figure S3). However, the mutant could no longer form aerial hyphae with *β*‐keto SCB2 fed at 40 h after inoculation (Figure 4D). These results are consistent with previous studies that timing is critical for A‐Factor's switching function^35^. There is a specific A‐Factor‐sensitive period in the middle of the exponential growth, after which the exogenous addition of A‐Factor can no longer induce morphological differentiation under solid and liquid conditions.

## 3 DISCUSSION

We developed a microfluidic chip that achieved a nanogap‐stabilized liquid–air interface for single‐spore cultivation and lifecycle observation of *Streptomyces*. Two model strains (*S. coelicolor* and *S. griseus*) were cultivated in the chip at single‐cell/spore resolution under different nutrient conditions. Compared with other methods, our chip can achieve single‐cell long‐term cultivation and dynamic observation using sub‐nanoliter microwells and air chambers. Although other devices, such as μ‐dish, have been used to capture Streptomycetes growth on solid media^36^, the chip used in this study allows air permeability while maintaining the hyphae within a narrow microscopic focal range to facilitate whole lifecycle observation at high spatial resolution. Moreover, the chip can be easily disassembled for further in situ SEM imaging to reveal subcellular structures such as hydrophobic protein patterns on aerial hyphae. The chip's main channel can controllably supply nutrients and stimulants in a controlled manner. We can readily improve the throughput of the chip by increasing the number of microwells with extended channels. Besides, we may use it to investigate the cell–cell interaction between *Streptomyces* and pathogenic bacteria by serial loading and cocultivation of *Streptomyces* and pathogens.

The whole lifecycle differentiation is essential for studying morphogenesis and secondary metabolism of *Streptomyces* ^35^. Currently, morphological differentiation is mainly studied on‐solid plates because the surface of liquid culture is unstable and cannot support the growth of aerial hyphae and spores. Using our chip, we found that the early development of aerial hyphae is not necessarily correlated with nutrient depletion as traditional solid‐based cultivation studies have suggested. The *S. griseus* Δ*afsA* mutant showed similar differentiation phenomena under solid culture and chip‐based liquid environments. Our chip provides higher spatial resolution and long‐term stability. Furthermore, we successfully restored the wild‐type phenotype of *S. griseus* Δ*afsA* mutant by adding *β*‐keto SCB2. Using the chip, we can also study effects of various molecules on morphological differentiation and secondary metabolism with significantly reduced reagent consumption by virtue of miniaturization.

Overall, we anticipate that the nanogap‐stabilized microfluidic chip will provide a new platform for studying *Streptomyces* development under precisely controlled microenvironments at the single‐cell level. Previous studies on the differentiation of *Streptomyces* in liquid media mainly focused on the analysis of pellet and clump formation^37^, which affects the production of secondary metabolites of *Streptomyces* such as *S. coelicolor* ^17^ and *S. noursei* ^38^. We envision that our chip can help establish the developmental model of other *Streptomyces* strains in liquid culture, which will be beneficial for optimizing industrial fermentation. Having *Streptomyces*' complete lifecycle on the microfluidic chip may also awaken cryptic gene clusters for the secretion of secondary metabolites and lead to the discovery of novel antibiotics for combating the global antimicrobial resistance crisis.

## 4 MATERIALS AND METHODS

### 4.1 Bacterial strains and materials

The microbial strains used in this study include *S. coelicolor* M145, *S. griseus* IFO 13350, and *S. griseus* Δ*afsA* mutant. These strains were cultured on the Mannitol‐Soy agar plate at 28°C for about a week to allow spore germination. The spores were harvested by sterile cotton swabs and suspended in the sterilized culture medium. The suspension was filtered through a filter tube filled with cotton wool to remove aerial hyphae. The OD_600_ of the spore suspension was adjusted to 0.15 to ensure that most microwells contain either one or zero spores. MM and YEME media were used for on‐chip cultivation.

### 4.2 Fabrication of the chip

The microfluidic chip was made of two glass plates and fabricated by standard photolithography and wet chemical etching techniques^39^. The photomask was designed using AutoCAD and ordered from MicroCAD photomask Co. Ltd. The top plate has a 55‐μm‐deep channel, with 40 microwells symmetrically distributed along the channel with a volume of 280 pl. The bottom plate consists of an array of nanopatterns of 1.5 μm in height (Figures 1C and 2A). The top plate has two access holes drilled by a diamond drill bit 0.8 mm in diameter. The glass plates were cleaned with ethanol, oxidized in a plasma cleaner, and silanized with 1*H*,1*H*,2*H*,2*H*‐perfluorooctyl trichlorosilane.

### 4.3 Device operation and cell cultivation

The glass chip was thoroughly cleaned with ethanol and tightly clamped by clips. The spore suspension was aspirated into a pipette and loaded into the channel leading to the microwells (Figure 1B). The suspension in the channel was aspirated from the outlet to remove excess spores to prevent channel block caused by hyphae growth, but the microwells could retain liquid medium and spores. Two syringes were connected to the chip by Teflon tubing to infuse the culture medium continuously for long‐term cultivation. The chip was placed under an inverted microscope to capture pictures every hour. A CO_2_ microscope cage incubator was placed around the microscope to maintain the temperature at 28°C for *Streptomyces* cultivation.

### 4.4 On‐chip SEM imaging

When cultivation terminated, the chip was transferred to a freezer at −20°C for 1 min to freeze the sample so that the hyphae could not move when we opened the chip. The chip was disassembled quickly, and the top plate was cut into 0.5× 0.7 cm pieces and fixed in 3% glutaraldehyde overnight at 4°C to maintain the bacterium's morphology. The sample was then washed with deionized water to remove glutaraldehyde and dehydrated in a series of ethanol solutions (50%, 70%, 85%, 95%, and 100%). After critical point drying (EM CPD300; LEICA) and gold coating (E‐1045), SEM was performed using HITACHI SU8018 (Hitachi).

### 4.5 Synthesis of A‐Factor analog *β*‐keto SCB2

*S. coelicolor* butanolides are *γ*‐butyrolactones from *S. coelicolor*, and *β*‐keto‐SCB2 is a stereoisomer of A‐Factor^40^, ^41^. The synthesis of *β*‐keto‐SCB2 began with the transformation of methyl 3‐oxocyclobutane‐1‐carboxylate to methyl 5‐oxotetrahydrofuran‐3‐carboxylate by the Baeyer‐Villiger oxidation^42^, ^43^, ^44^, ^45^. Methyl 5‐oxotetrahydrofuran‐3‐carboxylate was then reduced to 4‐(hydroxymethyl)‐dihydrofuran‐2(3*H*)‐one by the addition of NaBH_4_ followed by the protection of hydroxyl group with *tert*‐butyldimethylsilyl (TBS) chloride. Octanoyl chloride was slowly added to react with 4‐(((*tert*‐butyldimethylsilyl)oxy)methyl)dihydrofuran‐2(3*H*)‐one to give 4‐(((*tert*‐butyldimethylsilyl)oxy)methyl)‐3‐octanoyldihydrofuran‐2‐(3*H*)‐one. We then removed the silyl protecting group with tetrabutylammonium fluoride to afford *β*‐keto‐SCB2. Mass spectrometric analyses characterized the synthetic products. Detailed synthetic steps are described in Supporting Information: Methods.

## AUTHOR CONTRIBUTIONS

Yihua Chen and Wenbin Du conceived and designed the experiments, authored or reviewed manuscript drafts, and approved the final draft. Mengyue Nie, Wei Tang, and Yuwei Zhang performed the experiments. Dongwei Chen, Mengyue Nie, Yihua Chen, and Wenbin Du analyzed the data, prepared figures and movies, and wrote the manuscript. All authors contributed to the revision of the article.

## ETHICS STATEMENT

There is no animal or human research involved in this study.

## CONFLICT OF INTERESTS

The authors declare no conflict of interests.

## Supporting information

Supporting information.

Supporting information.

Supporting information.

Supporting information.

## Data Availability

Data supporting the findings of this study are available from the corresponding author upon reasonable request.
