# Supplementary material for: Whole lifecycle observation of single‐spore germinated Streptomyces using a nanogap‐stabilized microfluidic chip
Source: mLife. 2022 Sep 24;1(3):341–9. doi: 10.1002/mlf2.12039 (PMC10989842; doi:10.1002/mlf2.12039)
Supplement: Supplementary file 1 — Supporting information. [file MLF2-1-341-s002.docx]

**Supporting Information**

**Single-spore Germinated Whole Lifespan Observation of *Streptomyces* Using a Nanogap-stabilized Microfluidic Chip**

Dongwei Chen^1^, Mengyue Nie^1^, Wei Tang^1^, Yuwei Zhang^1^, Jian Wang^1^, Ying Lan^1^, Yihua Chen^1,2*^, Wenbin Du^1,3*^

^1^State Key Laboratory of Microbial Resources, Institute of Microbiology, Chinese Academy of Sciences, Beijing 100101, China

^2^College of Life Sciences, University of the Chinese Academy of Sciences, Beijing 100049, China

^3^Savaid Medical School, University of the Chinese Academy of Sciences, Beijing 100049, China

*Correspondence: Wenbin Du, wenbin@im.ac.cn; Yihua Chen, chenyihua@im.ac.cn

**SUPPORTING METHODS**

**Construction of the** ***Streptomyces griseus* (*S. griseus*) Δ*afsA* mutant**

The *idgS*-based blue-white screening system was used to construct the *S. griseus* ∆*afsA* mutant^1^. To eliminate the polar effects, we disrupted the *afsA* gene along with the following gene *bprA* that was involved in the A-Factor analogue biosynthesis (**Figure 4A**). Two 1.3-kb homologous arms flanking the *afsA* and *bprA* gene were obtained with the *S. griseus* IFO13350 genomic DNA as a template with the following primers afsA-LU (5’-CTTCCATGGGCACGCCCTAGGGCGCCTTCACCCGCTG ACT-3’ with a *BlnI* site shown by bold letters), afsA-LD (5’-AGCGGTATCCAGGGGCCTA GGGCACCATCTCGATCCCCAC-3’), afsA-RU (5’-GGGACTCTGG GGTTCCAATTGTGGTGCCCACGGTCCAAGA-3’ with a *MunI* site shown by underlining), and afsA-RD (5’-CTT GCTAGCAGATGTCAATTGGCCGAGGATGTTTACCCAGGTC-3’). The fragments were then cloned to the *MunI* and *BlnI* sites of pCIMt004 through a ligation-independent cloning strategy, resulting in pCIMt004_*afsA*_*bprA* (**Figure 4B**). The construction of pCIMt004_*afsA*_*bprA* was verified by nucleotide sequencing. Plasmid pCIMt004_*afsA*_*bprA* was then introduced into *S. griseus* IFO 13350 by *E. coli-Streptomyces* conjugation. After the blue-white screening, the apramycin-resistant double-crossover mutants were picked up and confirmed by PCR with an *afsA*-LU and *afsA*-RD primer pair (**Figure 4C**).

**Synthesis of A-Factor analogue *β*-keto SCB2**^2-7^

**(I) Methyl 5-oxotetrahydrofuran-3-carboxylate**

A dichloromethane (3 mL) solution of methyl 3-oxocyclobutane-1-carboxylate (0.1 g) at room temperature was added to H_2_O_2_ (30%, 0.25 g) with a few drops of acetic acid. The reaction mixture was then warmed to 50 °C overnight. The reaction mixture was quenched with saturated aqueous NaCl, extracted with dichloromethane, dried with Na_2_SO_4_, and concentrated under reduced pressure. The crude product was purified by silica gel chromatography to give methyl 5-oxotetrahydrofuran-3-carboxylate.

**(II) 4-(Hydroxymethyl)-dihydrofuran-2(3*H*)-one**

A THF (1 mL) solution of methyl 5-oxotetrahydrofuran-3-carboxylate (0.1 g) at 0 ℃ was added to NaBH_4_. A small volume of MeOH was added dropwise, and the reaction mixture was warmed to room temperature for 15 min. The reaction mixture was quenched with saturated aqueous NH_4_Cl, extracted with dichloromethane, dried with Na_2_SO_4_, and concentrated under reduced pressure. The crude product was purified by silica gel chromatography to give 4-(hydroxymethyl)- dihydrofuran-2(3*H*)-one.

**(III) 4-(((*tert*-Butyldimethylsilyl)oxy)methyl)dihydrofuran-2(3*H*)-one**

To a solution of 4-(hydroxymethyl)-dihydrofuran-2(3*H*)-one (20 mg) in 3 mL dichloromethane, TBSCl (38.8 mg), Et_3_N (100 mg), and catalytic amounts of DMAP were added. After stirring for 36 h, the reaction mixture was quenched with saturated aqueous NaCl, extracted with dichloromethane, dried with Na_2_SO_4_, and concentrated under reduced pressure. The crude product was purified by silica gel chromatography to give 4-(((*tert*-butyldimethylsilyl)oxy)methyl)dihydrofuran-2(3*H*)-one.

**(IV) 4-(((*tert*-Butyldimethylsilyl)oxy)methyl)-3-octanoyldihydrofuran-2-(3*H*)-one**

A solution of 4-(((*tert*-butyldimethylsilyl)oxy)methyl)dihydrofuran-2(3*H*)-one (40 mg) in THF (2 mL) was stirred at -78 ℃ for 5 min. LiHMDS (1 M in THF 0.3 mL) was then added dropwise to the reaction mixture and stirred for 30 min; octanoyl chloride (40 mg) was slowly added to the mixture and then warmed to room temperature overnight. The reaction mixture was quenched with saturated aqueous NaCl, extracted with dichloromethane, dried with Na_2_SO_4_, and concentrated under reduced pressure. The crude product was purified by silica gel chromatography to give 4-(((*tert*-butyldimethylsilyl)oxy)methyl)-3-octanoyldihydrofuran-2-(3*H*)-one.

**(V) A-Factor** **analogue,** **β-keto SCB2**

To a solution of 4-(((*tert*-butyldimethylsilyl)oxy)methyl)-3-octanoyldihydrofuran-2-(3*H*)-one in THF and acetic acid (1:1), TBAF (1 M in THF) solution was then added to the reaction mixture under ice-bath condition and then warmed to room temperature until the reaction was completed. The reaction mixture was quenched with saturated aqueous NH_4_Cl, extracted with dichloromethane, dried with Na_2_SO_4_, and concentrated under reduced pressure. The crude product was purified using silica gel chromatography to give *β*-keto SCB2 (2-[1′-hydroxyoctyl]-3-hydroxymethylbutanolide).

HR-ESI-MS spectrum of synthetic products are shown below:


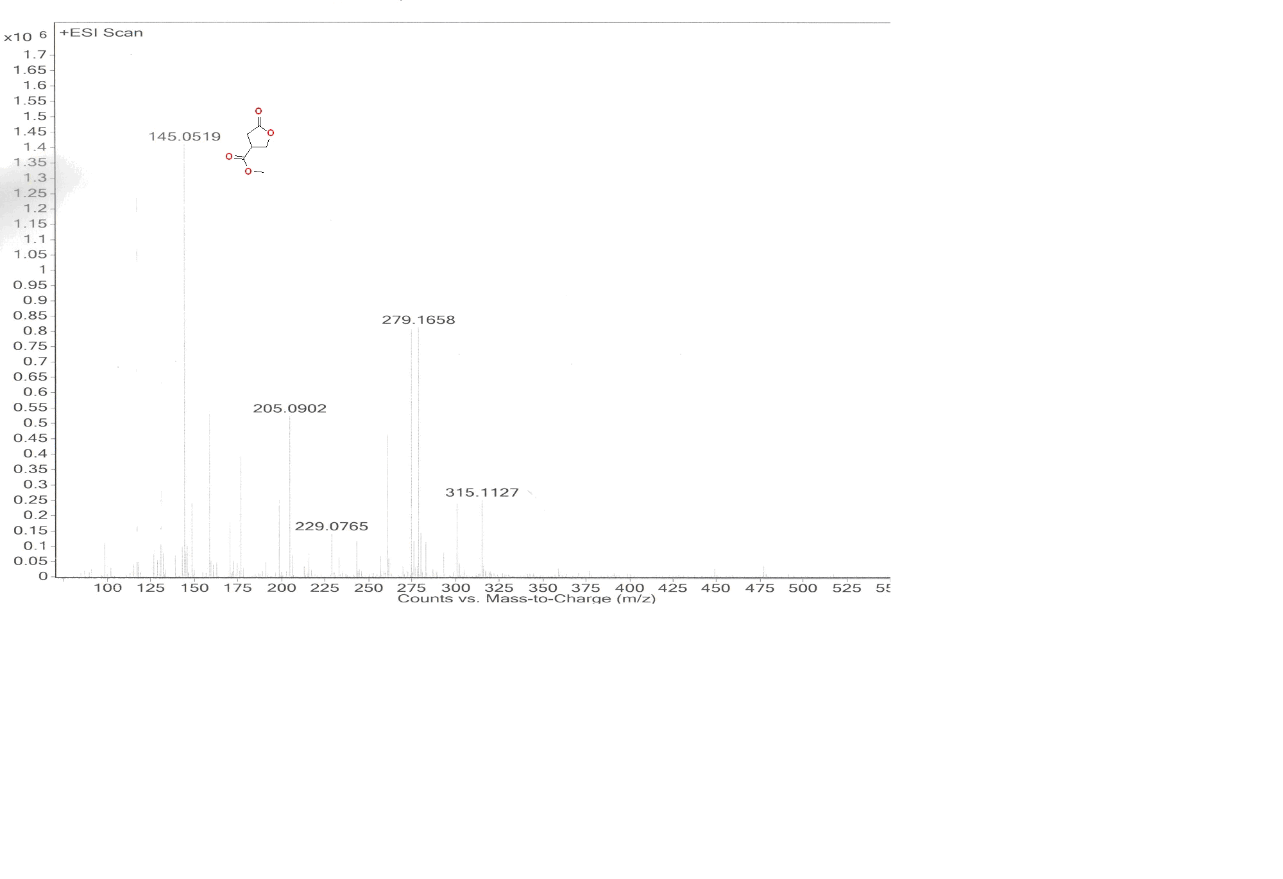


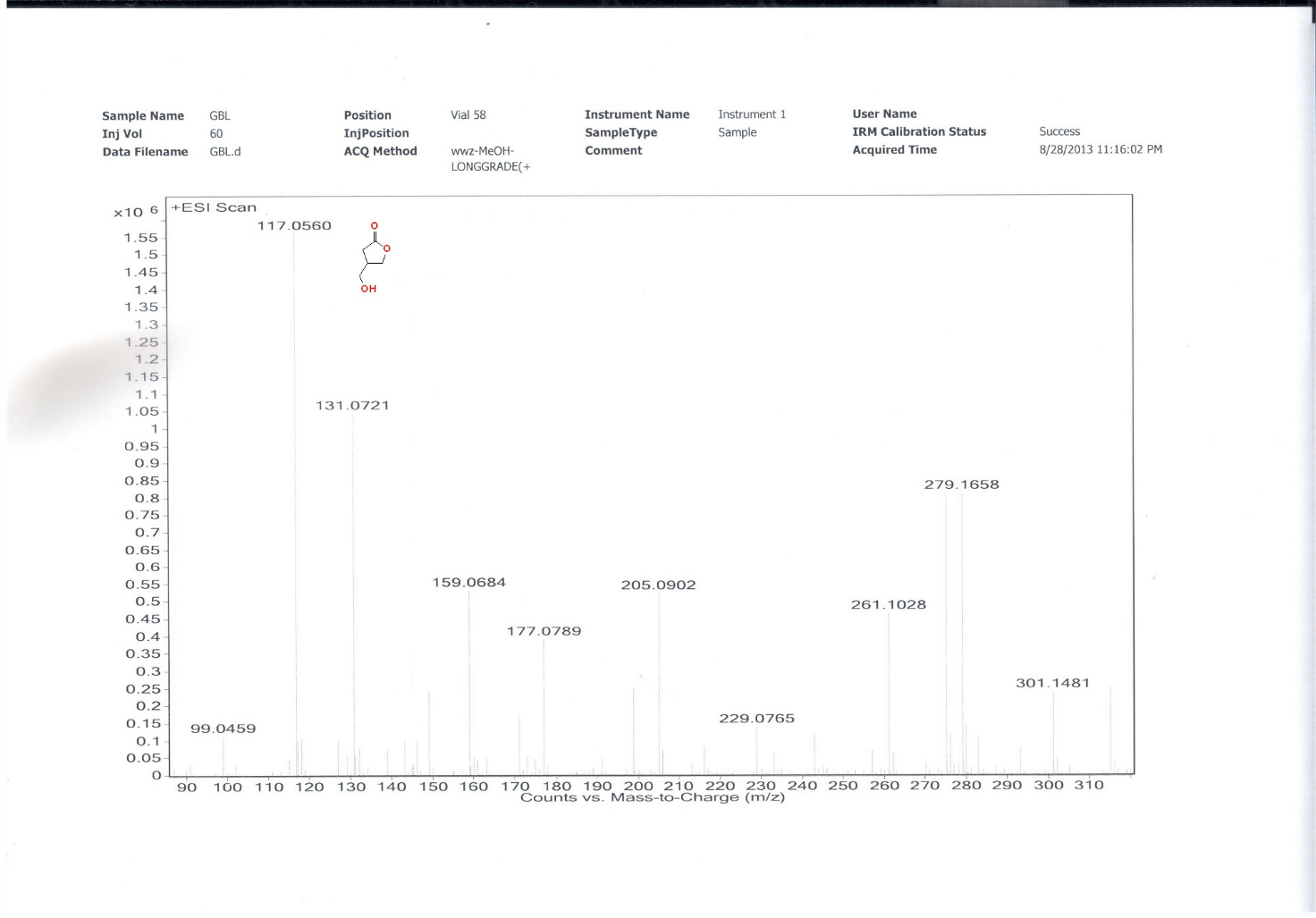


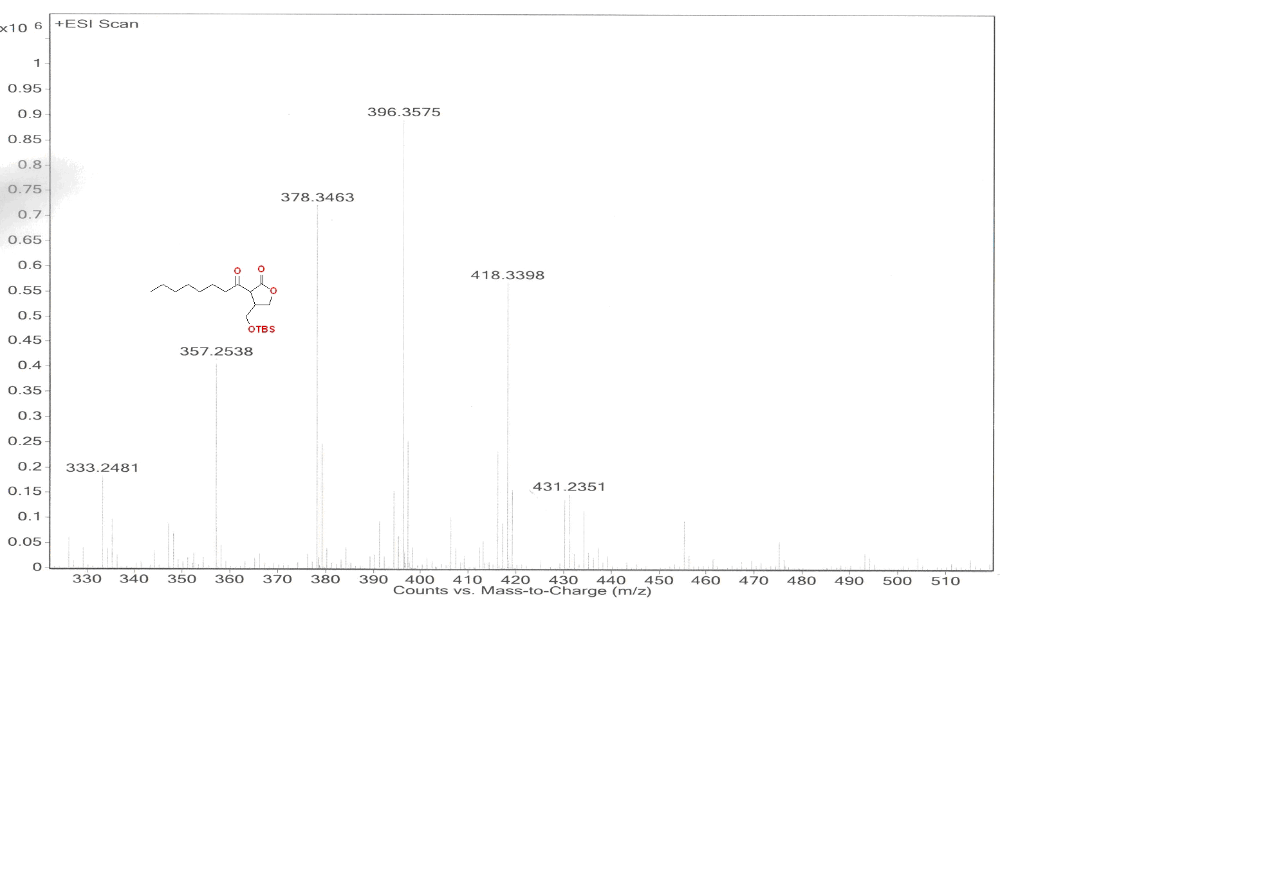


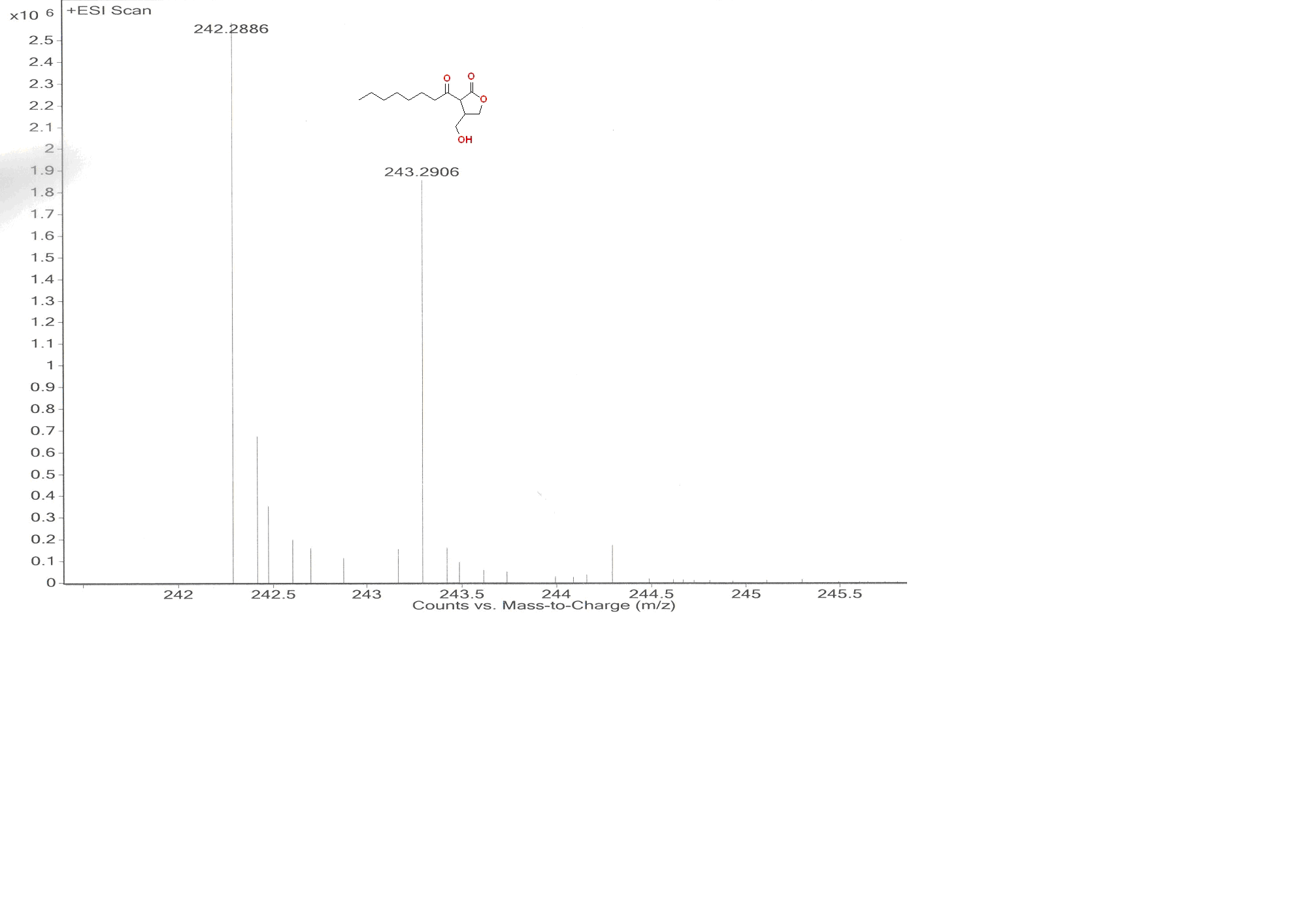


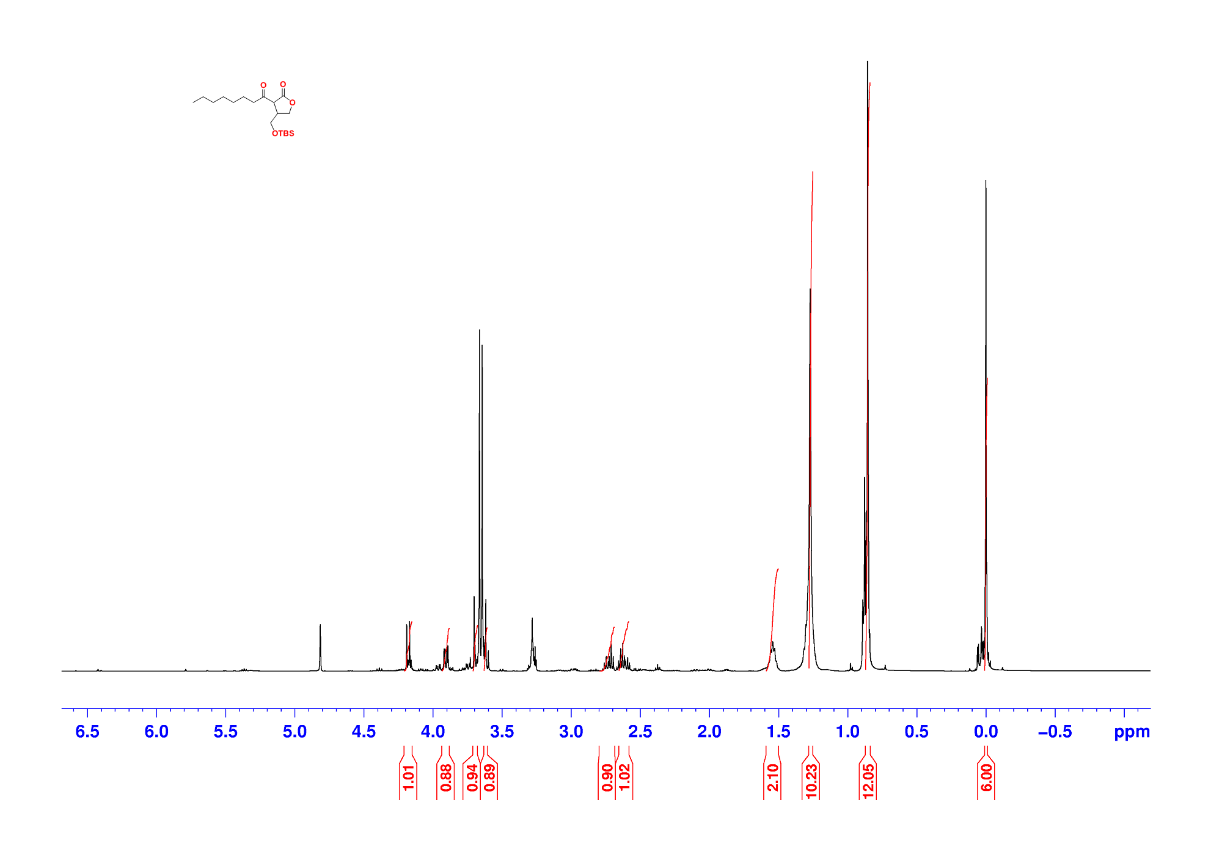


**REFERENCES**

1 Li P, Li J, Guo Z, Tang W, Han J, Meng X, et al. An efficient blue-white screening based gene inactivation system for *Streptomyces*. *Appl Microbiol Biotechnol*. 2015;99(4):1923-33.

2 Mori K, Chiba N. Synthetic microbial chemistry, XXIII. Synthesis of optically active virginiae butanolides A, B, C, and D and other autoregulators from streptomycetes. *Liebigs Ann Chem*. 1990(1):31-37.

3 Hsiao N-H, Nakayama S, Merlo ME, de Vries M, Bunet R, Kitani S, et al. Analysis of two additional signaling molecules in *Streptomyces coelicolor* and the development of a butyrolactone-specific reporter system. *Chem Biol*. 2009;16(9):951-60.

4 Parsons PJ, Lacrouts P, Buss AD. Synthesis of optically active A-factor. *J Chem Soc, Chem Commun*. 1995(4):437-38.

5 Chavan SP, Pasupathy K, Shivasankar K. Short and efficient synthesis of (±)‐A‐Factor. *Synth Commun*. 2004;34(3):397-404.

6 M. Crawforth J, Fawcett J, J. Rawlings B. Asymmetric synthesis of A-factor. *J Chem Soc, Perkin Trans 1*. 1998(10):1721-26.

7 Sarkale AM, Kumar A, Appayee C. Organocatalytic approach for short asymmetric synthesis of (*R*)-paraconyl alcohol: Application to the total syntheses of IM-2, SCB2, and A-Factor *γ*-butyrolactone autoregulators. *J Org Chem*. 2018;83(7):4167-72.

**SUPPORTING FIGURES**


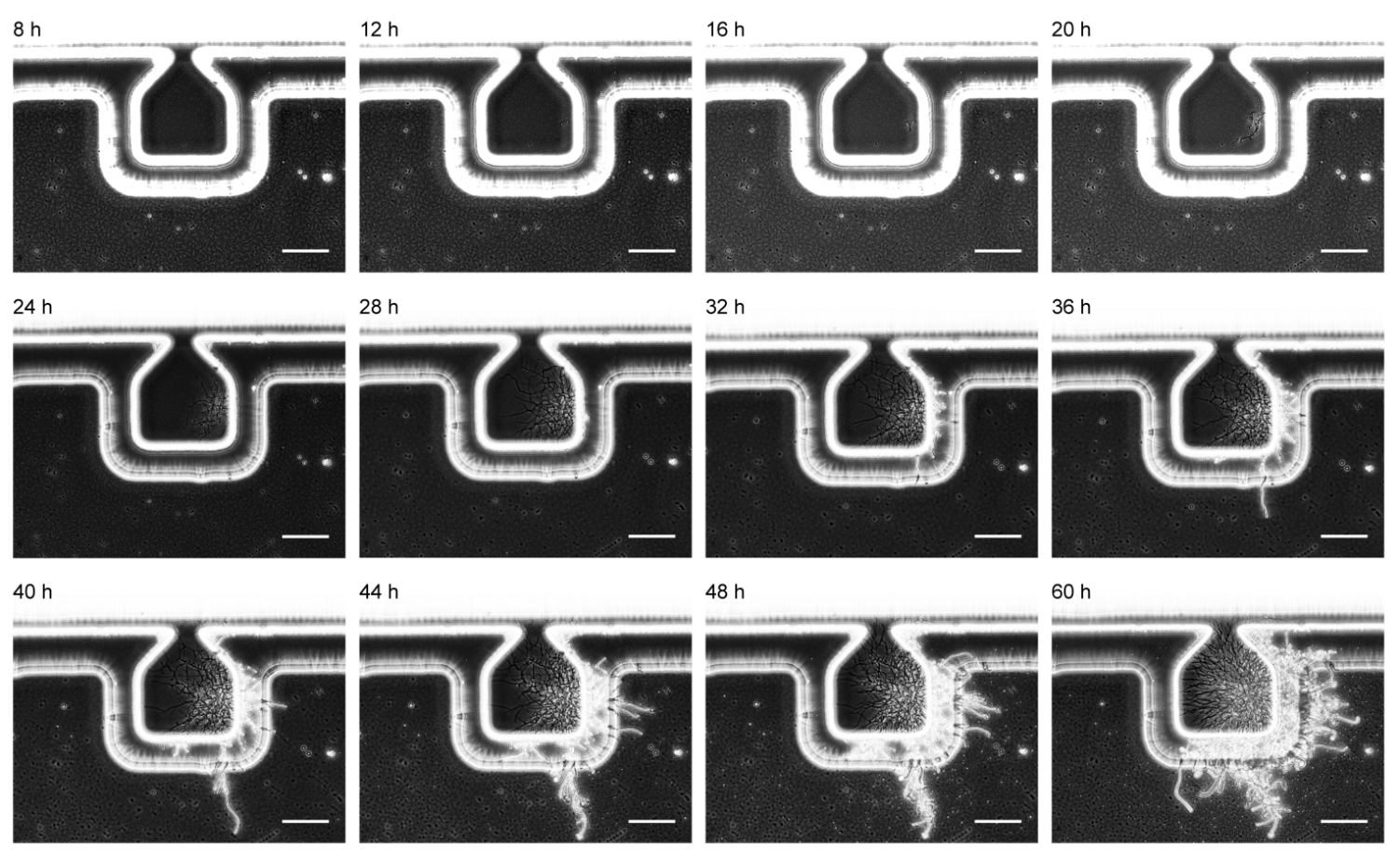


**Figure S1.** On-chip observation of lifespan development of *Streptomyces coelicolor* germinated from a single spore.


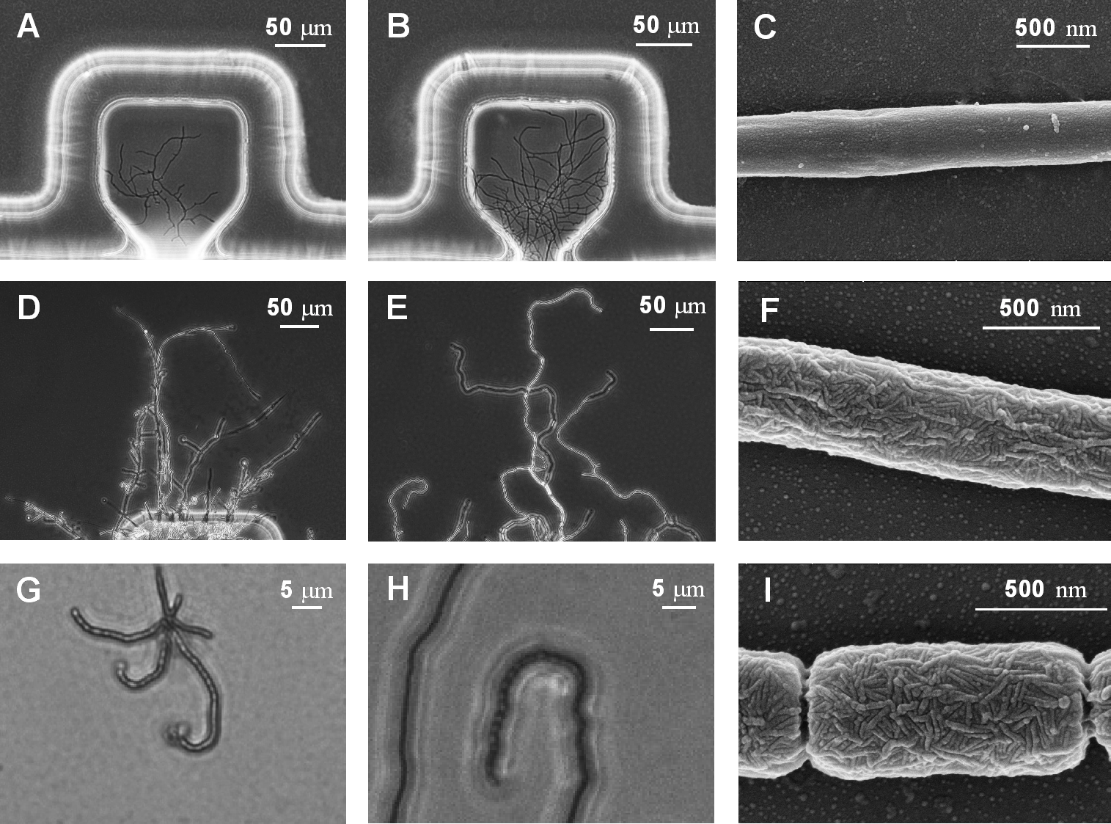


**Figure S2.** Images of different development stages of *Streptomyces griseus*. (A) Optical microscopy of vegetative hyphae cultivated with liquid MM media (B) Optical microscopy of vegetative hyphae cultivated with liquid YEME media. (C) Electron microscopy of vegetative hyphae. (D) Optical microscopy of aerial hyphae cultivated with liquid MM media. (E) Optical microscopy of aerial hyphae cultivated with liquid YEME media. (F) Electron microscopy of aerial hyphae. (G) Optical microscopy of spore cultivated with liquid MM media. (H) Optical microscopy of spores cultivated with liquid YEME media. (I) Electron microscopy of spores.


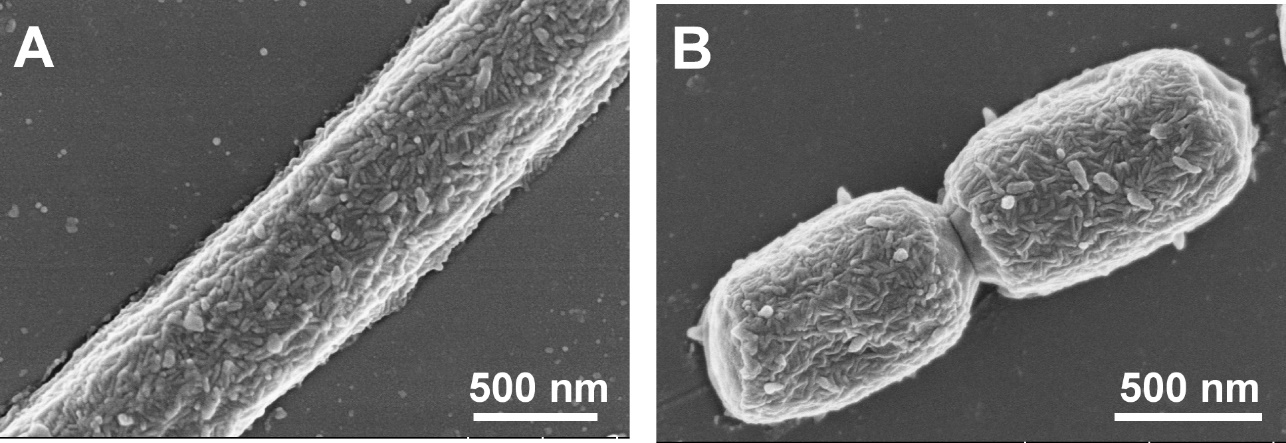


**Figure S3.** Aerial hyphae and spores observed from *Streptomyces griseus* Δ*afsA* mutant on device with A-Factor analogue supplied at 30 hours after initial cultivation. Representative electron microscopy of aerial hyphae (A) and spores (B).
