# Supplementary material for: Whole lifecycle observation of single‐spore germinated Streptomyces using a nanogap‐stabilized microfluidic chip
Source: mLife. 2022 Sep 24;1(3):341–9. doi: 10.1002/mlf2.12039 (PMC10989842; doi:10.1002/mlf2.12039)
Supplement: Supplementary file 2 — Supporting information. [file MLF2-1-341-s003.pdf]

streptomyces development chip 3.1

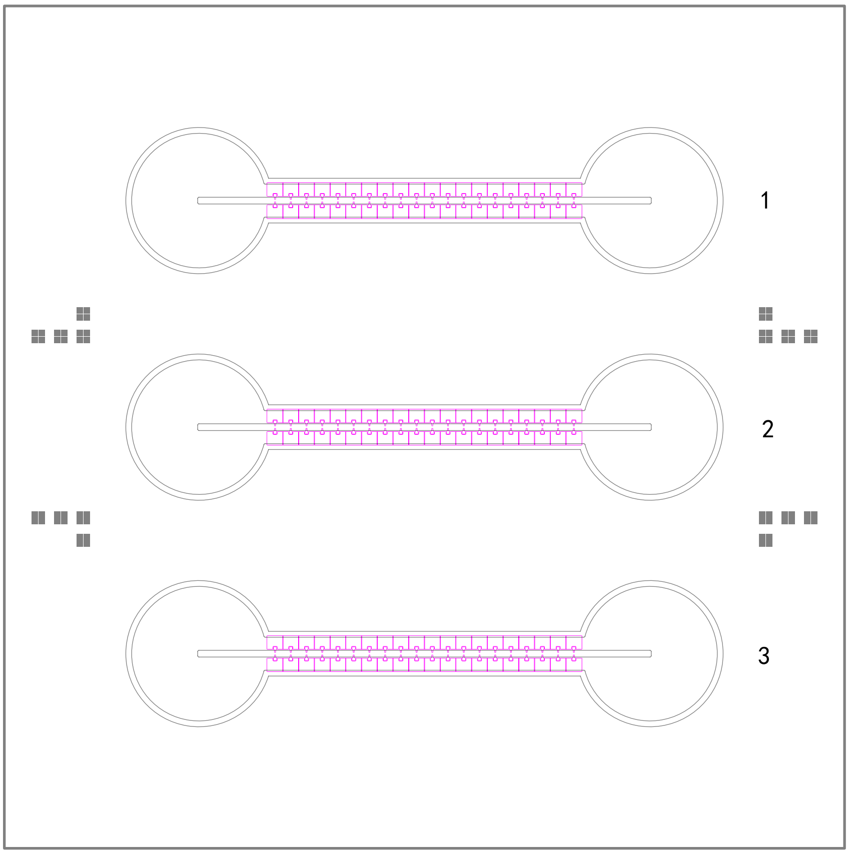

top plate: channel 55 um depth

top plate: microwells 15 um depth

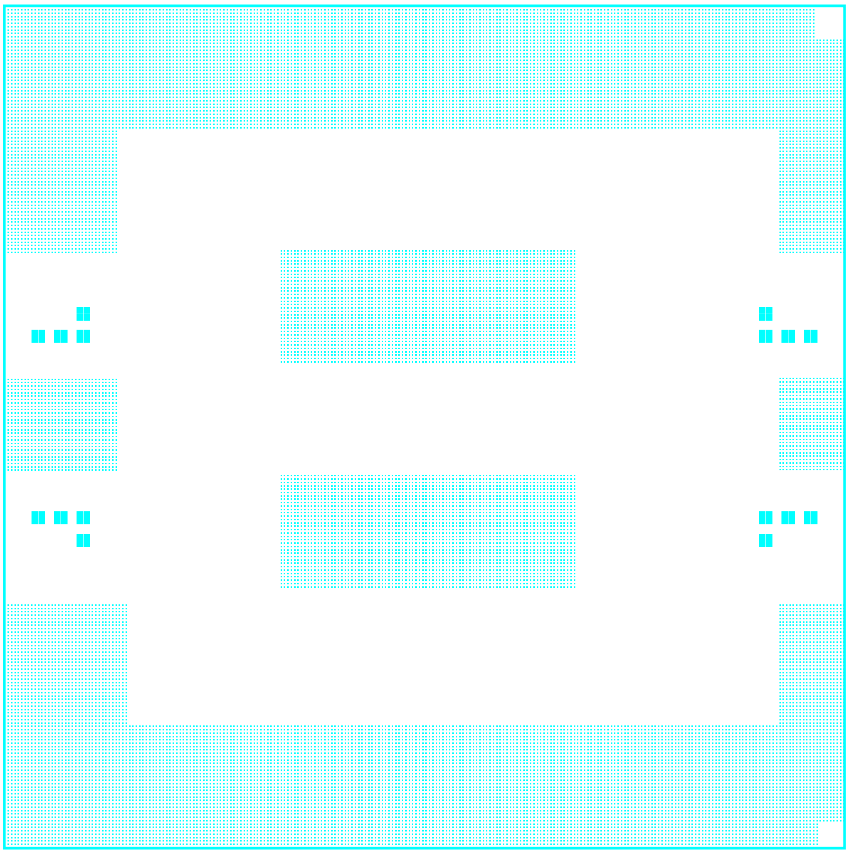

Bottom plate: nanopatterns 1.5 um height
chip size: 37.45 mm * 37.5 mm
